# Recent advances in near-infrared II imaging technology for biological detection

**DOI:** 10.1186/s12951-021-00870-z

**Published:** 2021-05-10

**Authors:** Nan-nan Zhang, Chen-ying Lu, Min-jiang Chen, Xiao-ling Xu, Gao-feng Shu, Yong-zhong Du, Jian-song Ji

**Affiliations:** 1 Key Laboratory of Imaging Diagnosis and Minimally Invasive Interventional Research of Zhejiang Province, Lishui Hospital, Zhejiang University School of Medicine, Lishui, 323000 Zhejiang China; 2 Institute of Pharmaceutics, College of Pharmaceutical Sciences, Zhejiang University, Hangzhou, 310058 China

**Keywords:** Second near-infrared (NIR-II) window, Fluorescence imaging, Biomedical applications

## Abstract

Molecular imaging technology enables us to observe the physiological or pathological processes in living tissue at the molecular level to accurately diagnose diseases at an early stage. Optical imaging can be employed to achieve the dynamic monitoring of tissue and pathological processes and has promising applications in biomedicine. The traditional first near-infrared (NIR-I) window (NIR-I, range from 700 to 900 nm) imaging technique has been available for more than two decades and has been extensively utilized in clinical diagnosis, treatment and scientific research. Compared with NIR-I, the second NIR window optical imaging (NIR-II, range from 1000 to 1700 nm) technology has low autofluorescence, a high signal-to-noise ratio, a high tissue penetration depth and a large Stokes shift. Recently, this technology has attracted significant attention and has also become a heavily researched topic in biomedicine. In this study, the optical characteristics of different fluorescence nanoprobes and the latest reports regarding the application of NIR-II nanoprobes in different biological tissues will be described. Furthermore, the existing problems and future application perspectives of NIR-II optical imaging probes will also be discussed.
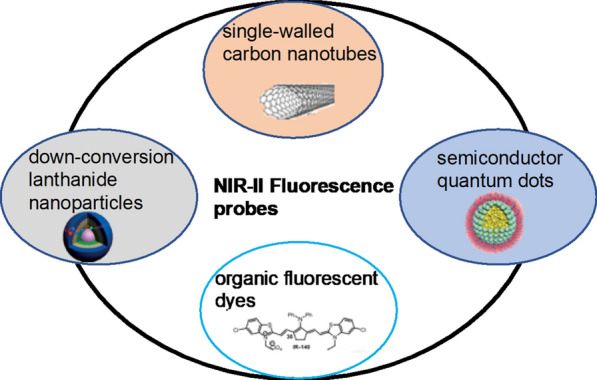

## Introduction

Medical imaging technology has promoted the understanding of the basic biological process based on anatomical or functional evaluations. This technology has enabled doctors to understand the disease pathways and diagnose the disease as early as possible. Various medical examination imaging technologies, such as computed tomography (CT), magnetic resonance imaging (MRI), and ultrasound imaging, can show biological anatomy information about the tissue or the organ. Other modalities include positron emission tomography (PET) and single-photon emission CT (SPECT), displaying biological function and molecular information for disease diagnosis. However, all of the clinically predominant technologies for disease diagnosis lack sensitivity and specificity, and some have potential radiological risks [1].

Optical imaging technology has emerged as an excellent noninvasive, real-time, highly sensitive and spatial resolution medical examination method for visualizing and detecting biological structures or events at the cell or tissue level [2–5], which meets the current requirements of clinical medicine. Optical signals can display various chemical components in the tissue, thereby providing useful functional information [6]. Conventional biological optical imaging approaches, such as photoacoustic (PA) and luminescence imaging, are mainly operated in the first near-infrared (NIR-I) region of the visible (400–700 nm) and near-infrared wavelengths (700–900 nm) [1, 7–9]. However, when imaged in the visible region (400–700 nm), they suffer from severe tissue scattering, absorption and autofluorescence, leading to a decreased spatial resolution, imaging sensitivity and contrast. [10–13].

To overcome these limitations, imaging in the second near-infrared window (NIR-II, 1000–1700 nm) has generated escalating interest. It has also been seen as the next-generation optical imaging technology used to assist diagnosis in biomedicine. Compared with NIR-I optical imaging, NIR-II optical imaging can reduce tissue photon absorption, tissue autofluorescence and scattering, permitting higher fidelity and spatial resolution effects [14]. Therefore, many efforts have been made to design and develop novel fluorescent probes that emit in the NIR-II window, including rare-earth (RE)-doped NPs [15–18], quantum dots (QDs) [19–22], organic fluorescent NPs [23–26] and single-walled carbon nanotubes (SWCNTs) [27–30]. Indeed, many of these probes have shown great application potential in biomedical examination-assisted diagnosis and surgical treatment. Therefore, the development of these fluorescent probes provides an essential choice for realizing the full potential of NIR-II optical bioimaging.

Notably, NIR-II biological imaging probe types and characteristics have been reported in detail in some recently published reviews [10–12]. Unlike these reviews, this article summarizes the current studies regarding NIR-II probe applications in imaging various biological tissues and diagnosing different diseases. First, the biological imaging characteristics of NIR-II probes and their advantages will be elaborated. Second, the application of NIR-II probes in different biological organs and tissues will be discussed. Finally, we address the current challenges and future application prospects of NIR-II bioimaging in biomedicine.

## NIR-II probes and their biological imaging properties

Excellent NIR-II fluorescence imaging probes require superior chemical properties and optical imaging performance. Conventional biological optical imaging approaches are mainly operated in the NIR-I spectrum region of the visible (400–700 nm) and near-infrared wavelengths (700–900 nm), which have high tissue autofluorescence, absorption and a limited penetration depth [31–34]. However, in recent years, an increasing number of research results have shown that optical imaging in the NIR-I spectrum region is far from satisfactory, limiting its applications in the biomedical field. Therefore, tremendous attention has been devoted to the development and optimization of NIR-II optical imaging technology. Compared with traditional optical imaging in the NIR-I spectrum, the application of NIR-II optical imaging can achieve lower tissue autofluorescence, photon scattering and absorption, and has a higher signal-to-noise (S/N) ratio and deeper biological tissue penetration [35–38]. The design and preparation of new NIR-II probes have recently become the focused research direction in optical imaging. Furthermore, many research teams have successfully developed various NIR-II probes and applied them to biomedical imaging.

NIR-II fluorescence nanoprobes can be divided into the following categories: semiconductor quantum dots (QDs), single-walled carbon nanotubes (SWCNTs), downconversion lanthanide nanoparticles (DCNPs) and organic fluorescent dyes.

Conceptually, SWCNTs are a piece of pure graphene rolled into a tube with a diameter of approximately 1 nm [39, 40]. SWCNTs have unique optical properties because of their photostability and large Stokes shifts of fluorescence emission in the NIR region [41–43]. At the same time, their absorption and autofluorescence of tissue or blood are minimal. When photons interact with SWCNTs, the energy will be absorbed and then released as fluorescence, and the emission wavelength range is 1000–1700 nm in the NIR-II window. However, their low photon-conversion efficiency is still a substantial obstacle, limiting the application of SWCNTs in fluorescence imaging.

QDs are semiconductor nanomaterials with a diameter of only 10–100 atoms. Compared with other fluorescent nanoprobes, QDs have excellent high fluorescence quantum efficiency and concentrated emission spectra [22, 44–46]. Nevertheless, at the same time, the potential biological toxicity of QDs hinders their application and development. Among the different kinds of QDs, Ag_2_S is widely used as an excellent theranostic nanoplatform due to its properties of NIR-II fluorescence and photothermal properties [21, 47]; it has lower biological toxicity than other QDs containing Te, Cd, Pb, As and Se, and the emission fluorescence spectrum is approximately 1294 nm in the NIR-II window [48]. Generally, QDs are comprised of metal elements, so unknown potential acute and chronic toxicity in vivo may be the main factor affecting their practical application.

Downconversion lanthanide nanoparticles (DCNPs) have become a promising fluorescent probe in the NIR-II window due to their narrow multipeak emission profiles, large Stokes shifts, deep soft tissue penetration and good photostability [49–52]. In principle, rare earth nanoprobes can produce fluorescence through the effect of downconversion between rare earth elements. Therefore, when excitation light irradiates the nanoparticles, it will emit fluorescence in the NIR-II window. Nevertheless, similar to other kinds of inorganic fluorescent probes, long retention in the body and potential acute or chronic toxicity may produce safety hazards and hinder their future translation [53, 54].

To date, organic fluorescent probes are still the preferred choice in optical imaging technology. Currently, two NIR probes (methylene and indocyanine green) are widely used in the clinic. From the perspective of practical biomedical points of view, organic fluorescent probes are progressively attractive because of their compact molecular structures, easy functionalization, high fluorescent quantum efficiency, minimal toxicity, availability for large-scale chemical synthesis and facile derivatization [55–57]. For example, semiconducting polymers are completely organic optical agents. Because they naturally avoid the toxicity problem caused by heavy ions, they have been widely used in NIR fluorescence [58], chemiluminescence [59, 60] and photodynamic therapy [61] research. However, the short wavelength in the NIR-I region will cause poor photon penetration. Therefore, it is necessary to develop organic probes with a high quantum yield, a low band gap and emission wavelengths in the NIR-II window. Some NIR-II organic dyes have been successfully synthesized, such as IR-1061 and CH1055 [62–64]. Although many organic fluorescent probes have been designed and synthesized for biosensing and bioimaging in deep tissue with high sensitivity and resolution, there are also some limitations in applying the materials. First, many organic dyes are not stable in aqueous solutions; second, biocompatible dyes with maximum emission wavelengths beyond 1200 nm are still lacking.

In general, SWCNTs have strong fluorescence intensity in the NIR-II window, enabling deep tissue penetration and high spatial resolution fluorescence imaging. However, the solubility and biocompatibility of SWCNTs are poor, which makes it impossible to directly use SWCNTs for bioimaging before modification. QDs show a high quantum yield and low photobleaching sensitivity. However, they tend to accumulate in the liver/spleen and are not easily excreted from the body.

DCNPs have received increasing attention due to their narrow and multimodal emission spectra, large Stokes shift and excellent photostability. However, similar to most of the reported NIR-II inorganic nanomaterials, their long residence time in the reticuloendothelial system and their inability to be removed from the living body increase their potential safety hazards.

Organic NIR-II probes can be effectively optimized in regard to their spectroscopic properties by adjusting their molecular structure, they are easily degraded, they have low biological toxicity, and they have great potential for in vivo imaging applications. However, they also have disadvantages such as a low quantum yield, poor water solubility, and poor stability in organisms that need to be further overcome.

## Application of NIR-II probes in biomedicine

Optical imaging technology is a noninvasive, efficient and safe inspection method. It is currently one of the most straightforward and versatile diagnostic modalities in the biomedical field. Therefore, some NIR-I probes have been used in various clinical theranostic procedures, such as vasculature imaging, fluorescein angiography, and tumor imaging and monitoring [65, 66]. However, due to the limited penetration depth of NIR-I probe-mediated optical imaging in the tissue (1–3 mm) and the obvious autofluorescence, traditional NIR-I probes are suboptimal for intraoperative imaging [67]. Understandably, NIR-II optical imaging has attracted increasing attention due to its negligible tissue autofluorescence and minimal photon absorption and scattering, providing unparalleled soft tissue penetration (a few centimeters) and spatial resolution (approximately 10 µm); these superior properties are expected to make it the first choice for assisting with disease diagnosis and guiding surgical procedures [1, 12, 68, 69].

## Cancer imaging and image-guided surgery

To date, surgical resection is still the most essential and standard treatment for tumors. Therefore, the ability to visualize the tumor tissue boundary and find the surrounding metastasis during tumor surgery will play a vital role in the treatment prognosis. Much progress has been made in tumor imaging research and in guiding tumor resection through optical imaging in the NIR-II window [16, 70, 71]. Wang et al. developed a novel NIR-II fluorescence imaging probe (DCNP) modified with targeted peptides and complementary DNA to achieve the accuracy of optical image-guided resection for metastatic ovarian cancer [16]. In this work, a novel complementary DNA-mediated self-assembly bioimaging strategy of nanomedicine in vivo was successfully developed. Furthermore, this work showed that the use of the DCNPs prepared in this study for NIR-II optical imaging was significantly better than the clinically used ICG. Another exciting result was its in vivo self-assembly bioimaging strategy. The administration by two sequential injections can achieve long-term retention of the drugs in the tumor tissues (6 h), which served as the optimal surgical time window. Meanwhile, this NIR-II probe and the in vivo self-assembly strategy also realized the visualization of metastatic lesions and guided the removal of ≤ 1 metastatic lesions. This novel optical imaging in the NIR-II window for image-guided tumor resection provides a method for practical clinical applications.

**Fig. 1:**
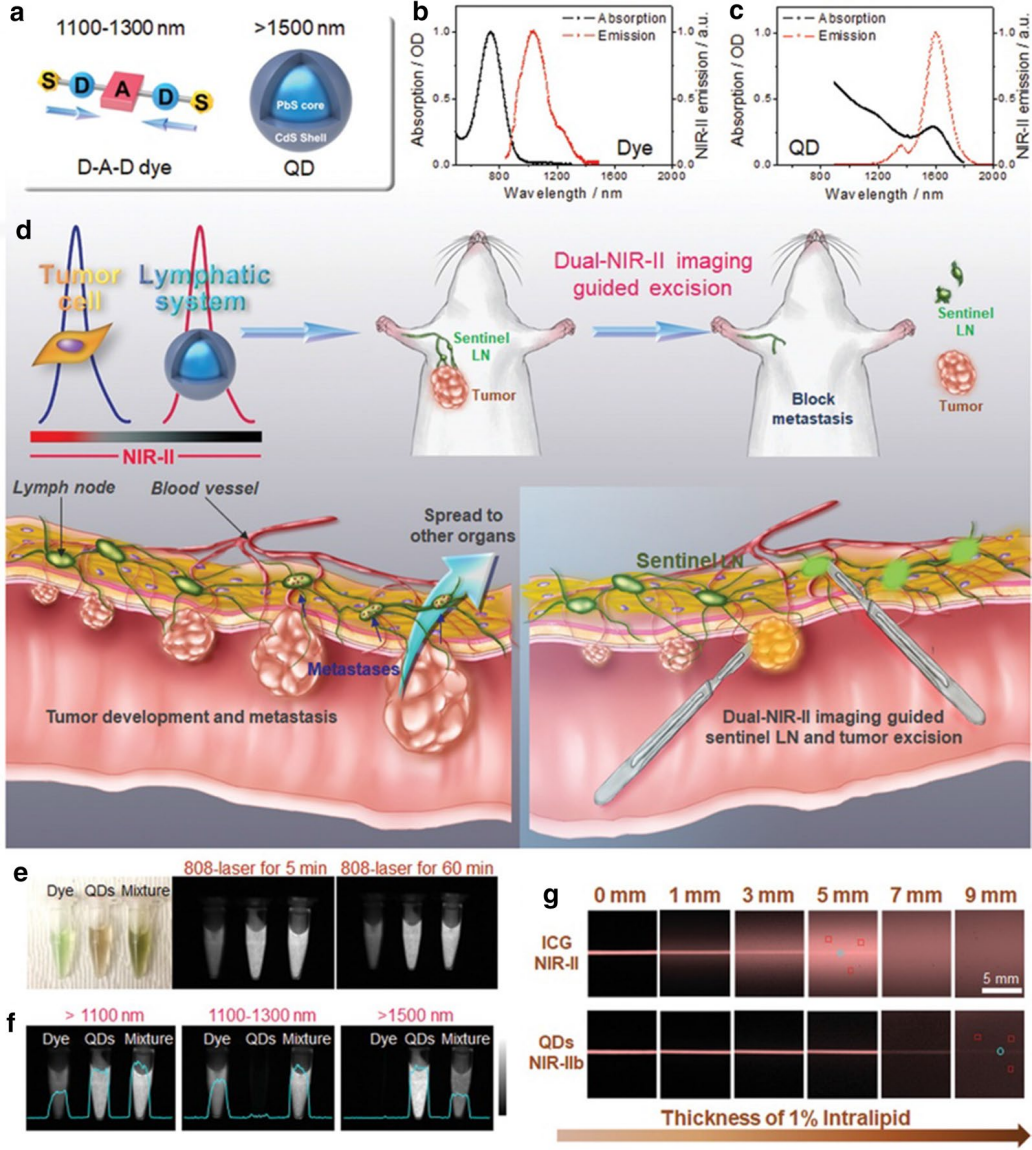
Dual-NIR-IIa and NIR-IIb fluorescence imaging-guided sentinel LN resection. **a**–**c** The optical properties of DAD dye and PbS QDs. **d** Schematic illustration of imaging-guided sentinel LN resection. The DAD probe was injected intravenously, and then the QDs were injected into the tumor. **e** The photostability of the DAD dye and PbS QDs. **f** Color testing of the dual-NIR-IIa and NIR-IIb probes. **g** Fluorescence images of QDs and ICG. [73]

The metastasis and spread of cancer cells mainly depend on the surrounding lymphatic vessels. Detecting the adjacent lymph nodes (sentinel LNs) can evaluate whether the cancer has metastasized [72]. The gold standard of biopsy for the detection and targeted removal of sentinel LNs is the preoperative use of technetium-99 m for lymphoscintigraphy. Therefore, it is urgent to look for a safer and more straightforward method of observing sentinel LNs to reduce the radiological hazards caused by lymphoscintigraphy. Tian et al. [73] reported a multiplexed NIR-II fluorescent nanoprobe with zero autofluorescence and significantly suppressed scattering, which enables the visualization of metastatic tumors and metastatic proximal LN resection. In this study, a dual NIR-II probe pair, a donor–acceptor–donor (D–A–D) fluorescent probe IR-FD, and PbS QDs were synthesized for NIR-II imaging. The synthesized D–A–D fluorescent probe IR-FD was used for fluorescence imaging of primary/metastatic cancer in the NIR-IIa spectrum (1100–1300), and PbS QDs were applied to detect cancer-invaded sentinel LNs in the NIR-IIb (> 1500 nm) window (Fig. 1a–d). This research has shown that IR-FD can increase the quantum yield in water (> 6.0%). At the same time, it also exhibited excellent optical imaging effects in vivo. Compared with the clinically used near-infrared tracer indocyanine green, the prepared QDs showed perfect photostability and brightness, and noticeable bleaching occurred after their exposure to a laser for 5 h (Fig. 1e, g). When using QDs for NIR-IIb imaging, they have low scattering, high contrast, and a minimal signal-to-muscle ratio to achieve high-quality LN imaging under indoor light, guiding the removal of sentinel LNs. This combination of dual-NIR-IIa and NIR-IIb fluorescence imaging technology has good clinical application potential because NIR-IIa optical imaging can locate the primary tumor. The NIR-IIb imaging channel can be applied to map the sentinel LNs so that NIR-IIb imaging-guided resection can be achieved under bright light, which is practical in clinical applications.

## NIR-II imaging for stem cell tracking

Stem cells have unlimited or immortal self-renewal ability and can be used to treat many incurable diseases, including liver diseases, heart failure, bone defects, immune system disorders, and cancer [74, 75]. After transplantation into the body, stem cells converge on damaged organs and then differentiate into target cells to apply their therapeutic function. Therefore, real-time monitoring of the status of transplanted stem cells in vivo is of extraordinary significance. In recent years, many NIR probes have been extensively researched for stem cell tracking in vivo.

**Fig. 2:**
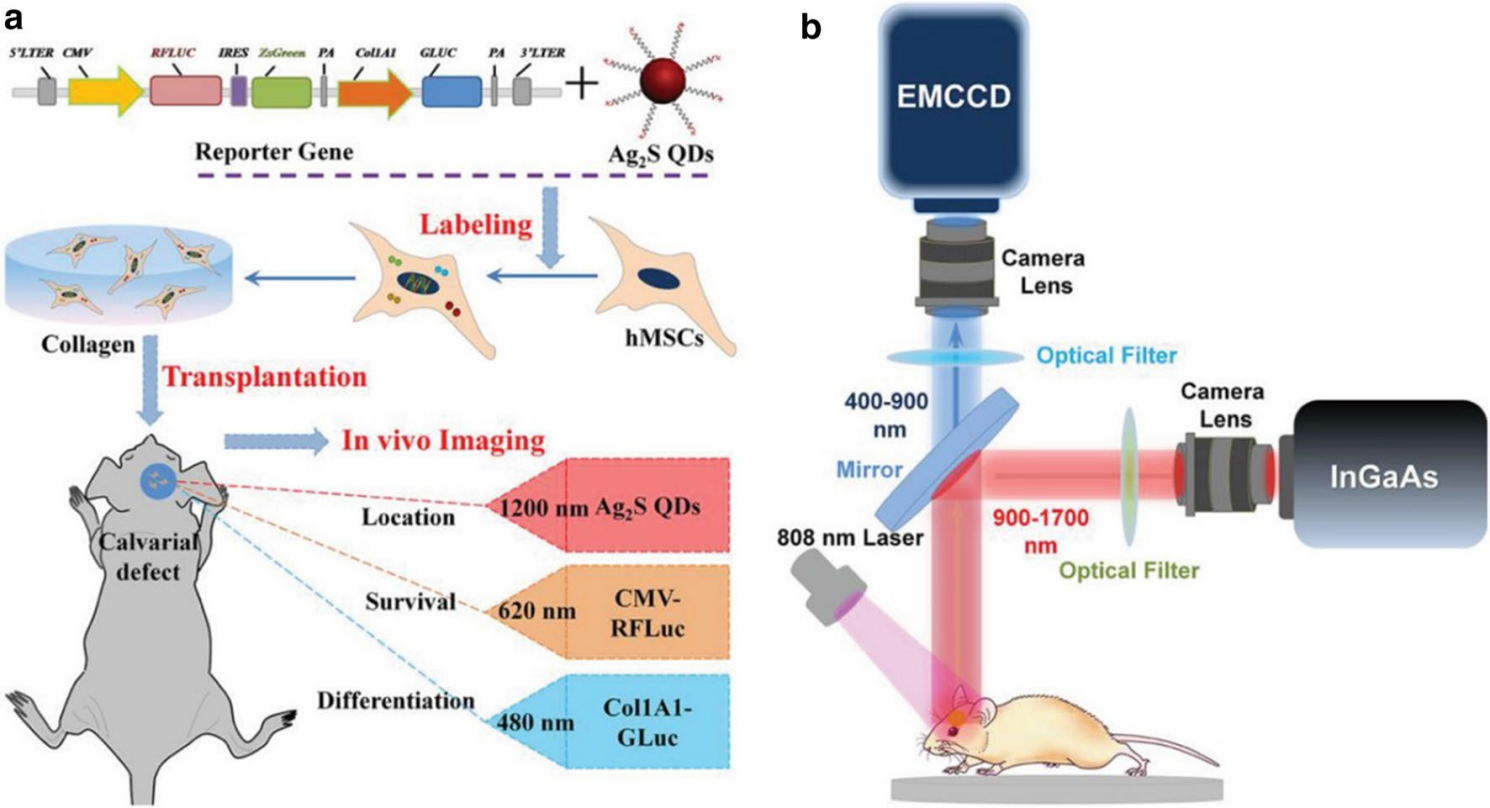
Scheme of the NIR-II fluorescence/dual bioluminescence multifunctional optical imaging system. **a** Schematic illustration of the multifunctional optical imaging strategy and **b** fluorescence imaging equipment with a 400–1700 nm wide spectral detection range in vivo [76]

In Wang Qiangbin’s paper (Huang et al. 2020) published in Advanced Functional Materials in 2019 [76], he reported NIR-II fluorescence/dual bioluminescence (BLI) multiple imaging technologies, covering the visible spectrum and NIR-II spectrum from 400 to 1700 nm, which can visualize the location, survival, and differentiation of transplanted human mesenchymal stem cells (hMSCs) in real time (Fig. 2a, b). In this study, prepared Ag_2_S QDs were used to visualize the distribution of transplanted hMSCs in the body, and red firefly and Gaussian luciferase-based BLI was applied to observe the survival and the osteogenic differentiation status of the hMSCs. NIR-II fluorescence imaging was performed on the experimental mice after treatment with Ag_2_S QDs. The fluorescence signal slightly decreased in the bone defect position at 30 days, indicating that hMSCs can stay in the lesion for a long time. They used the red firefly and Gaussian luciferase-based BLI imaging method to observe the differentiation status of hMSCs in the target tissue. Therefore, combining NIR-II fluorescence and bioluminescence (BLI) imaging technology, the process of hMSCs locating in the target position and then undergoing osteogenic differentiation and gradual apoptosis within 30 days after bone regeneration can be monitored. This novel multiple imaging techniques can significantly expand the multifunctional supervision of stem cell treatment efficiency and contribute to realizing the applications and clinical transformation of stem cells.

Yang et al. (2019) developed biocompatible PbS QDs for monitoring the cellular migration, biological distribution and clearance information of injected mesenchymal stem cells (MSCs) and used them to treat supraspinatus tendon tears in mice [77]. In this research, the prepared PbS QDs were modified with Tat peptide to label the QDs, enter the MSCs and locate the MSCs. The results showed that the transplanted MSCs had similar migration directions. More QD-labeled MSCs were enriched around the footprint and improved to 82% at 7 days postinjection in the medium-density group. In the stage of repair, the MSCs stayed in the footprint for the longest time with the highest cell retention rate. Therefore, the NIR-II optical imaging method based on PbS QDs had excellent performance in monitoring the status of MSCs in the body, facilitating the optimization of MSC therapy.

## NIR-II-responsive drug release

To date, smart nanoplatforms for on-demand drug release have been widely studied, as they enable precise control of the dose and position of the loaded drug, thereby minimizing side effects while maximizing the therapeutic efficacy [78]. These nanoplatforms can achieve on-demand drug release in response to some specific stimuli, such as pH, light, and biomolecules. Among the above stimuli, light has the properties of simple remote controllability, noninvasiveness, high temporal and spatial resolution, and minimal tissue damage [79]. Therefore, the nanoplatform based on the NIR light response has great potential in precision disease therapy.

**Fig. 3:**
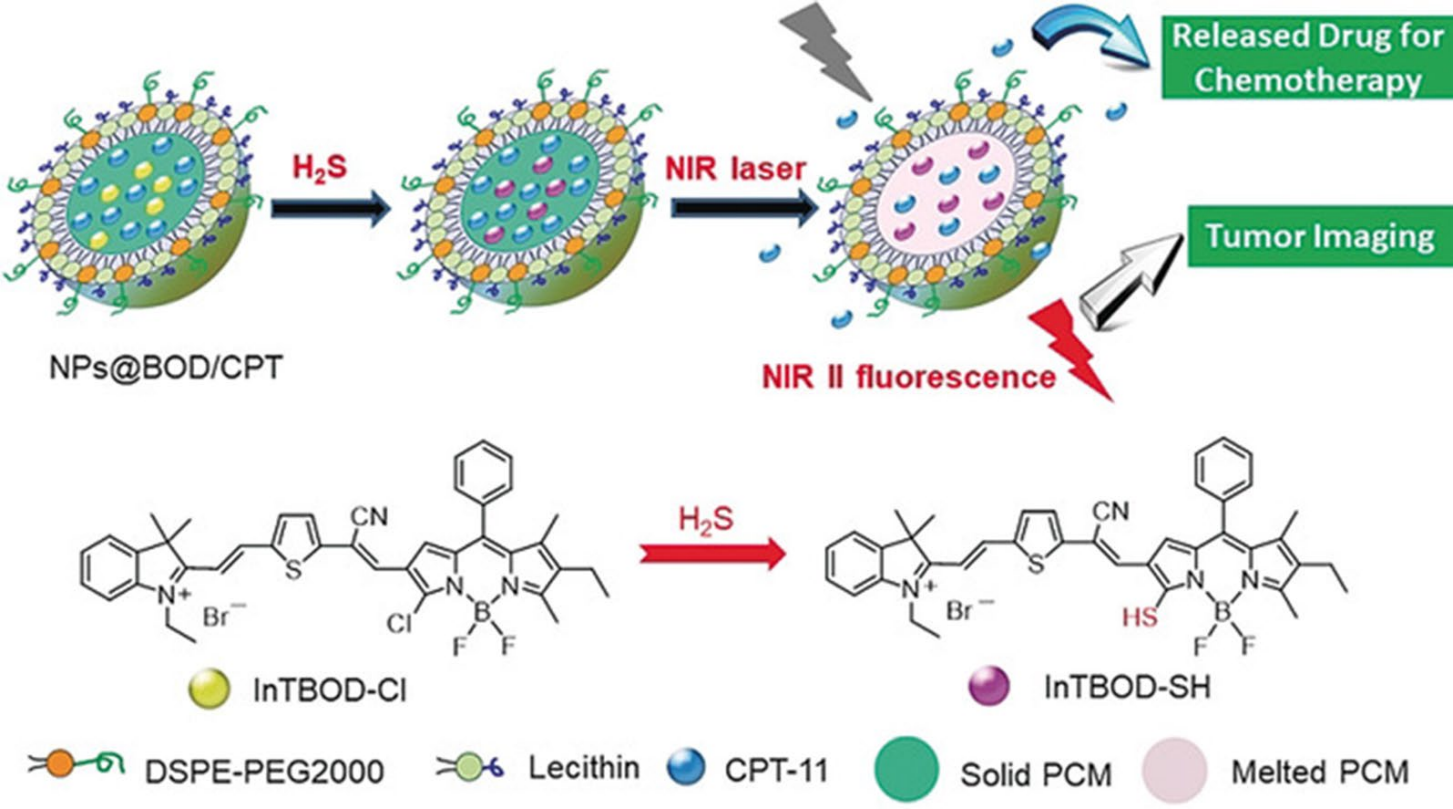
Scheme of NPs@BOD/CPT for tumor NIR-II fluorescence imaging and drug release [80]

Shi et al. [80] reported a theranostic nanoplatform (NPs@BOD/CPT). It was fabricated by boron-dipyrromethene (InTBOD-Cl) to endow it with H_2_S-triggered NIR light to achieve light-controlled on-demand drug release for cancer treatment. H_2_S can trigger the production of NIR photothermal agents. These photothermal agents convert light energy into heat, thereby raising the temperature above the eutectic melting point, causing a phase change and triggering the release of the loaded drug. Furthermore, H_2_S also activated bright NIR-II emission, enabling H_2_S-rich cancer diagnosis and photocontrolled on-demand drug release (Fig. 3). This nanoplatform targets H_2_S rich cancers. HCT116 tumor-bearing mice were treated with nanoparticles and irradiated with a laser. The tumor growth was significantly inhibited with minimal side effects, which indicated that the accumulated NPs@BOD/CPT in the tumor position could be activated specifically by NIR light. This theranostic nanoplatform provides new ideas for precision medicine under the guidance of NIR-II fluorescence imaging.

In addition, Huang et al. [81] proposed a novel strategy using NIR-II-responsive drug release in collaborative chemical/photothermal tumor therapy. In this study, bare InSe nanosheets (InSe NSs) with a thickness of approximately 5 nm were prepared and then PEGylated to improve their dispersion and stability in vivo. The nanoprobe exhibited a remarkable NIR-II photothermal conversion efficiency of 39.5%, photothermal stability and NIR-II response loaded drug release properties. In vitro cytotoxicity assays showed that InSe-DOX has higher cytotoxicity than free DOX at the same DOX concentrations. Almost all of the cells were killed after treatment with InSe-DOX at a low concentration of 10 µg/mL under irradiation. However, the PEGylated InSe NSs showed negligible NIR-II photothermal killing efficiency at the same concentration. These excellent synergistic chemo/photothermal effects of the prepared InSe-DOX have great potential in therapeutic applications in medicine.

## NIR-II imaging of inflammation

Inflammation is a common clinical symptom that can occur in many organs and tissues of the human body. Quantitative imaging of the distribution of inflammatory factors in living organisms is vital for understanding fundamental biological processes. Optical imaging in the NIR-II window has a higher penetration depth and contrast than NIR-I optical imaging, which can provide real-time monitoring of deep biological organs and tissues [82]. Therefore, NIR-II optical imaging has broad prospects for in vivo sensing and high-resolution imaging.

**Fig. 4:**
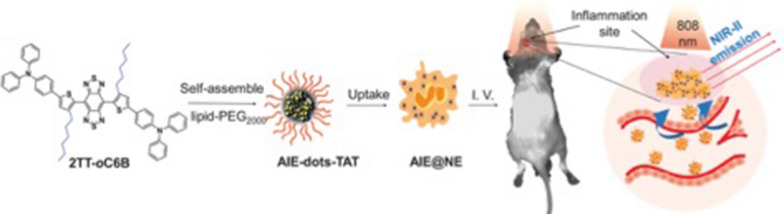
Scheme of the preparation of AIE@NE and its application for brain inflammation imaging [83]

**Fig. 5:**
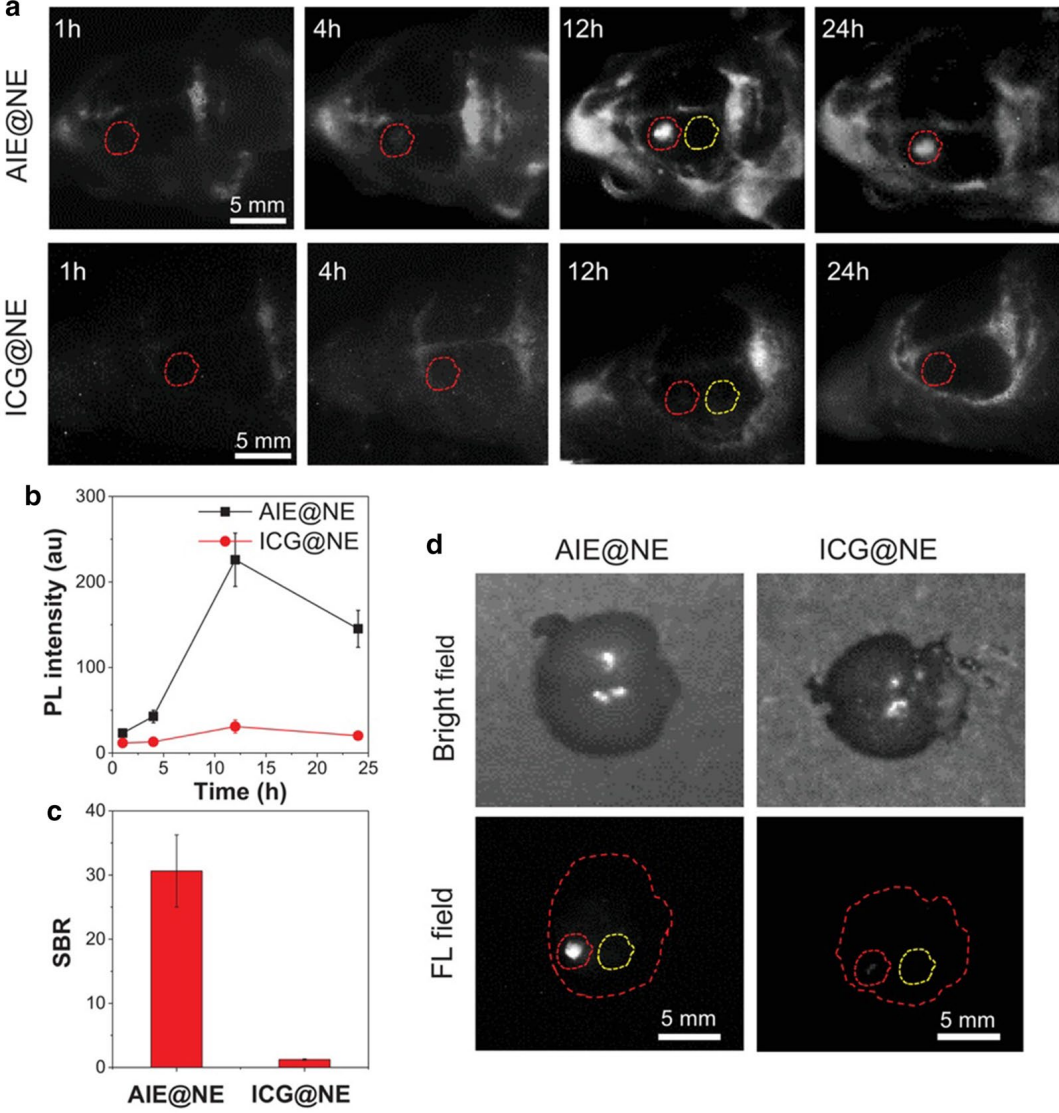
AIE@NE for brain inflammation NIR-II imaging. **a** Time-dependent NIR-II fluorescence imaging of brain inflammation. **b** The average fluorescence signal of the inflammatory area at different time points. **c** The SBR of the inflammatory area at 12 h. **d** Bright field and fluorescent field photos of the brain tissue [83]

Liu et al. [83] reported constitutional isomerization based on the philosophy of aggregation-induced emission (AIE). Based on the AIE molecular design principle, NIR-II and AIE nanoprobes (2TT-oC6B) with excellent performance were prepared by benzobisthiadiazole (acceptor), thiophene (bridge and donor), and triphenylamine (molecular rotor and second donor). The prepared probe 2TT-oC6B exhibited an emission peak at 1030 nm and a high quantum yield of 11%. To provide the AIE nanoprobe with a targeting imaging ability, neutrophils (NEs) were utilized to modify 2TT-oC6B (AIE@NE) to penetrate the brain tissue and then accumulate in the inflammatory tissue (Fig. 4). Furthermore, this study demonstrated that the NE-coated AIE nanoprobe could cross the BBB (blood–brain barrier) and be located in the inflammation site. Moreover, the signal-to-noise ratio was 30.6, which was higher than that of ICG (Fig. 5).

Other related research on the imaging of inflammation using the NIR-II probe includes a paper published in 2019 by Zhang's team about developing and applying a new 1550 nm emitting nanoprobe for detecting lymphatic inflammation [84]. NIR-II optical imaging technology has attracted considerable attention due to its deep penetration depth and high signal-to-noise ratio. Among the various NIR-II probes, those emitting in the NIR-IIb (1500–1700 nm) window can provide the lowest photon scattering based on the Mie theory [85]. However, one of the application limitations of the NIR-IIb probe is its response to biological stimuli. Based on the above situation, they developed a new nanoprobe that responds to reactive oxygen species (HOCl) for excellent fluorescence imaging of inflammation. The probe system was constructed between Cy7.5 and Er^3+^-doped DCNP according to the absorption competition-induced emission (ACIE), so it combined all of the advantages of DCNP and Cy7.5, such as the long wavelength fluorescence of Er^3+^-doped DCNP under excitation at 808 or 980 nm and the sensitive (500 nM), fast (within 1 min), and selective responsive of Cy7.5 to HOCl. The results showed that the 1550 emissive nanoprobe has a superior spatial resolution in a scattering tissue phantom with a penetration depth of 3.5 mm. A clear anatomical structure of lymphatic inflammation was observed in the ratiometric channel with a high resolution of ~ 477 µm.

## NIR-II imaging of tumor vessels

Tumor blood vessels play vital roles in the tumorigenesis, growth and metastasis of solid tumors. The abundant blood vessels around the tumor can deliver oxygen and nutrients to the tumor. Variations in tumor vasculature have been used for tumor staging evaluation and are considered an essential indicator for predicting the outcomes of tumor metastasis. The formation of tumor vasculature mainly depends on the specific biological characteristics of solid tumors, such as the tumor size, tumor model, and tumor site. Moreover, the changes in tumor blood vessels can also be used to reflect the effects of tumor treatment. Therefore, it is necessary to monitor tumor blood vessel formation and variation, providing practical guidance for tumor staging, treatment and the evaluation of tumor metastasis. The commonly used examination techniques for blood vasculature include B-mode ultrasonography, computerized tomography and magnetic resonance imaging (MRI), which usually provide information about the general tumor size, anatomical structure and its relationship with its surrounding tissues, but the dynamic structures and activities inside the tumors cannot be accurately probed [86]. Due to the reduced light scattering and absorption in biological tissues, NIR-II optical imaging has recently demonstrated attractive performance in tumor vascular imaging.

**Fig. 6:**
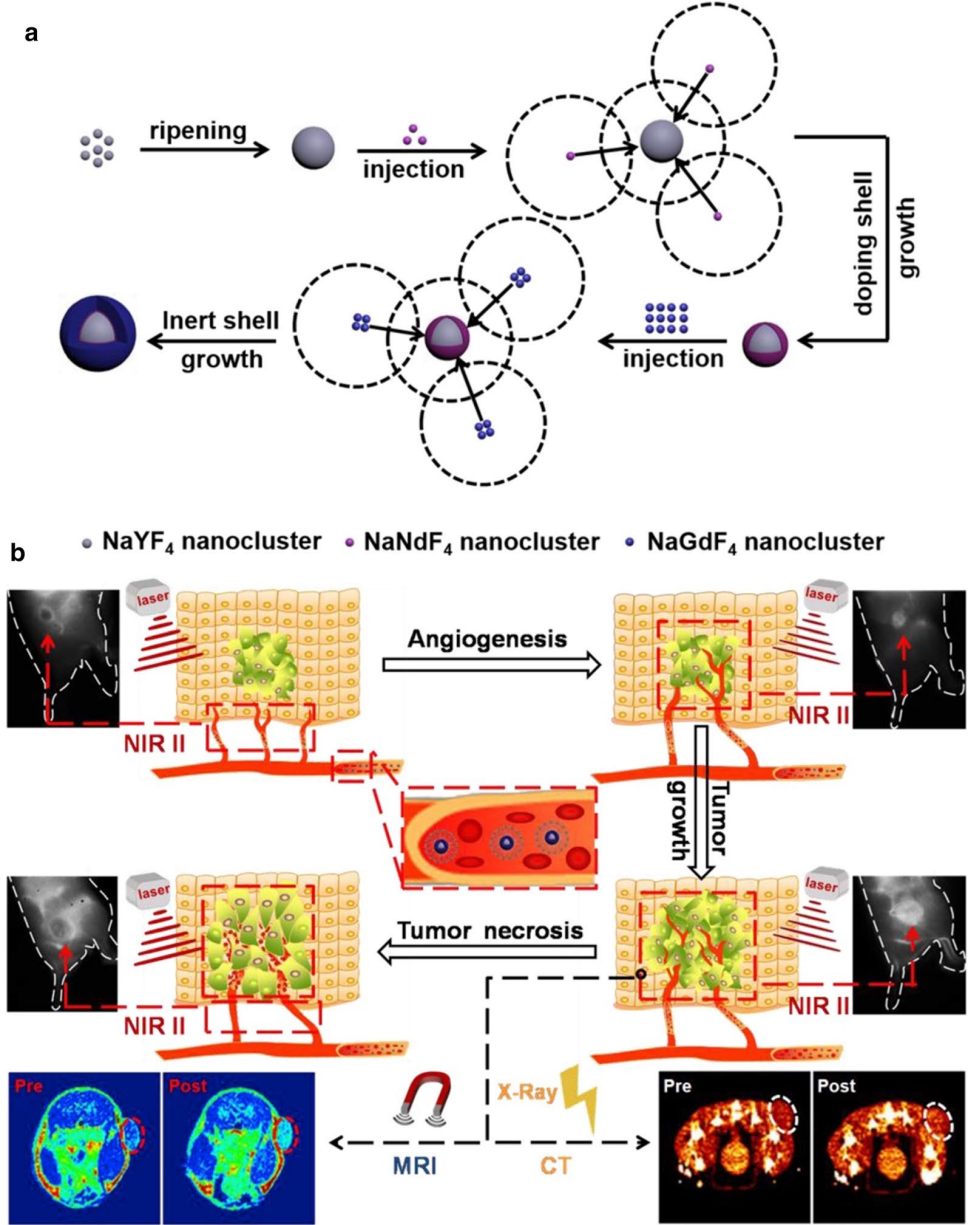
**a** Schematic illustration of the preparation of the NaYF_4_:5%Nd@NaGdF_4_ nanoprobe and **b** its application in NIR II, MRI and CT imaging of tumors during tumorigenesis, growth and necrosis [87]

Ren et al. reported a well-dispersed Nd^3+^-doped core–shell downconversion luminescent nanoprobe (NaYF4:5%Nd@NaGdF4), which has a long emission wavelength in the NIR-II window, fast attenuation to X-rays, and strong temperature-dependent paramagnetism [87]. NaYF_4_ nanoclusters were used as precursors for the core, dopant, and shell to prepare monodisperse Nd^3+^-doped core–shell nanoprobes with bright NIR-II luminescence through a one-pot reaction, as shown in Fig. 6a. Furthermore, the core–shell nanoprobe also exhibited magnetism and attenuation toward X-rays, which is helpful for improving MRI and CT imaging (Fig. 6b).

**Fig. 7:**
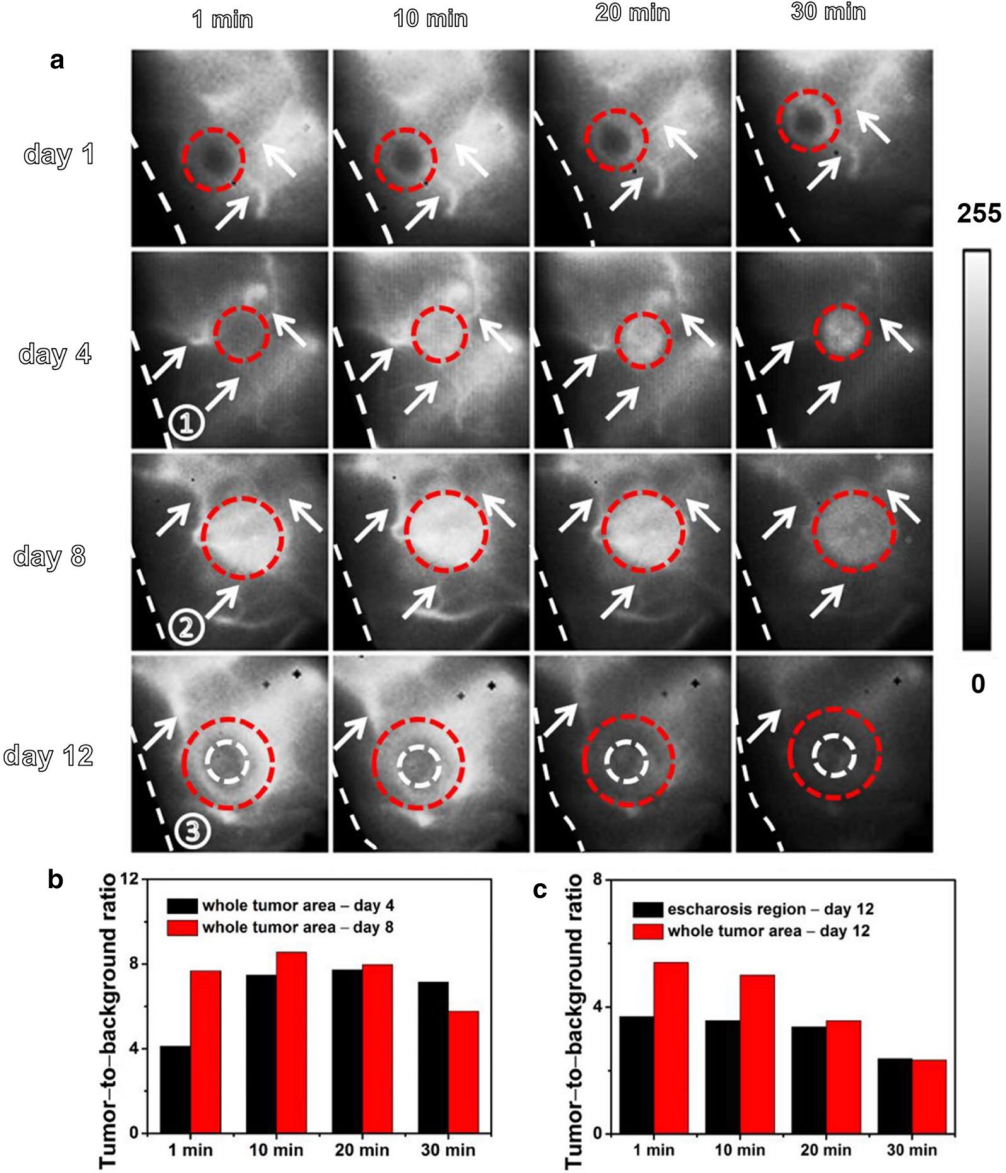
NIR II imaging monitors the changes in the tumor vasculature. **a** NIR II fluorescence imaging of tumors in a 4T1 breast tumor model. **b**, **c** The tumor-to-background ratios at different times [87]

After modifying the surface of the nanocrystals with DSPE-PEG_2k_, it exhibited prolonged blood circulation, good biocompatibility and biosafety in vivo. The nanoprobe showed a relatively high quantum yield (0.52%) compared to the fluorescence dye IR-26. Then, NIR-II imaging based on NaYF_4_:5%Nd@NaGdF_4_ successfully realized the visual monitoring of tumor blood vessels in breast tumor models. The formation and changes of tumor blood vessels during tumorigenesis, growth and necrosis were tracked (Fig. 7a). As the nanocrystals circulated in the body and gradually infiltrated into the tumor tissue (red dashed circle), the tumor gradually became bright, and 20 min after drug injection, the ratio of tumor-to-background steadily increased (Fig. 7b). The continuous decrease in the ratio of tumor-to-background indicated that the NaYF_4_:5%Nd@NaGdF_4_ nanocrystals were challenging to circulate and retain in the tumor, which may be due to the angionecrosis in the tumor and the formation of escharosis (Fig. 7c). In conclusion, this Nd-based NIR-II nanoprobe not only has potential in the monitoring of tumor progression, tumor staging and tumor therapy but it also has great potential in imaging the vasculature during tumorigenesis, thrombosis and atherosclerosis.

In another example, bright NIR-II-conjugated polymer nanoparticle (CP NP)-assisted optical-resolution photoacoustic microscopy imaging (ORPAMI) technology was used to pinpoint the vasculatures of the tumor and cerebral tissue [88]. The CP NPs were prepared by the microfluidics method and have the advantages of a small uniform size, high sensitivity, large extinction coefficient (48.1 L/g), excellent photoacoustic stability, and good biocompatibility. The sensitivity of PA was as high as 2 µg/mL. 3D ORPAMI can observe the normal vasculature of mouse ears in a wide field of view. The resolution was 19.2 µm, the SBR was 29.3 dB, and the maximum imaging depth was 539 µm. The tumor edge comprised of twisted dense blood vessels in the surrounding normal regular blood vessels can be depicted. Cerebral blood vessels can also be clearly distinguished through the intact skull with a high SBR (22.3 dB), high resolution (25.4 µm), and a large imaging area (48 mm^2^) at depths up to 1001 µm by using 3D ORPAMI technology. This study showed that NIR-II CP NP-assisted optimal-resolution photoacoustic microscopy imaging technology is promising for various biomedical applications.

## Conclusion and perspectives

In this article, we summarized the properties and characteristics of organic and inorganic NIR-II probes. We described recent studies regarding the application of these probes in NIR-II optical imaging in different biological tissues. The reported NIR-II probes, including DCNPs, QDs and organic dyes, have demonstrated excellent performance in fluorescence imaging in different biological tissues (Table 1). Optical imaging in the NIR-II window permits more in-depth penetration and higher resolution because of lower tissue scattering and autofluorescence than traditional optical imaging in the visible region or the﻿NIR-I window. Therefore, excellent optical imaging by the NIR-II window can serve as a powerful tool for clinical applications.

**Table 1:** Representative NIR-II nanoprobes fabricated for biomedical applications

	Fluorescence probe	Excitation/emission wavelength	Application	Quantum yield (%)	References
Quantum dots	PbS QDs	808/ > 1500 nm	Imaging of metastatic tumor and proximal LNs resection	N.A.	[73]
		808/ 1300 nm	Monitoring the cellular migration, biological distribution and clearance information of injected mesenchymal stem cells	17.3	[77]
	Ag_2_S QDs	808/ 1200 nm	Tracking the location, survival, and osteogenic differentiation of transplanted human mesenchymal stem cells	15.5	[76]
Down-conversion NPs	NaGdF_4_: 5% Nd@NaGdF_4_	808/ 1060 nm	In vivo self-assembly bioimaging to improve the image-guided surgery for cancer	N.A.	[16]
	Er^3+^-doped DCNP	808 and 980/ 1550 nm	High-resolution ratiometric sensing of lymphatic inflammation	0.27–2.73	[84]
	NaYF_4_:5%Nd@NaGdF_4_	808/ 1060 nm	Visualizing the formation and variation of tumor blood vessels	0.52	[87]
Organic NPs	NPs@BOD/CPT	810/ 900 nm	Photo-controlled on-demand drug release for cancer therapy	0.012	[80]
	2TT-oC6B	808/ 1030 nm	Imaging of the brain inflammation tissue	11	[83]
	Conjugated polymer nanoparticles	1064 nm/ 1160 nm	3D vasculature imaging in vivo	N.A.	[88]

With the rapid development of nanomedical science, an increasing number of fluorescence probes will be designed, studied and applied. However, it is worth noting that NIR-II optical imaging technology is still in its primary stage. Many prepared NIR-II nanoprobes in reported papers have some problems and challenges in biological applications. To realize the extensive application of NIR-II optical imaging technology in biomedicine, it is necessary to address the current problems.

First, many organic NIR-II nanoprobes are not stable in water because the conjugative backbone can be attacked by reactive species such as water. Furthermore, the emission wavelength of many existing organic dyes is only slightly higher than 1000 nm, and they have low fluorescence quantum yields, only approximately 0.3–2%. Efforts should be made to reduce the bandgap of the probes to the bathochromic shift wavelength, which maintains its stability in aqueous solutions.

Second, one should fully understand the biocompatibility, metabolic pathways and long-term toxicity of the NIR-II nanoprobes in the body. For example, lanthanide-doped nanoparticles displayed many desirable properties, such as a long luminescence lifetime, large Stokes/anti-Stokes shifts, multiple emission bands, and narrow emission bandwidths in many reported studies. However, few studies have examined the pharmacokinetics or the acute or chronic toxicity of these probes in vivo. Crucially, only when an NIR-II nanoprobe has excellent biocompatibility and acceptable clearance characteristics (similar to the clinically approved NIR-I dyes, such as methylene blue and ICG) can clinical approval and translation be achieved. Therefore, it is necessary to clarify the biological toxicity of the probe and its metabolic pathways in the body. The design and development of a highly biocompatible and safe NIR-II probe will have unprecedented practical value.

Third, improving the quantum yield (QY) of nanoprobes is very important. This is an indicator to evaluate the optical performance of fluorescence probes. The higher the QY of the fluorescent dye, the longer the fluorescence lifetime. Therefore, a high QY can make a fluorescent probe emit a strong fluorescence signal, which can effectively improve the sensitivity of optical imaging. However, most NIR-II probes suffer from low QY, especially in an aqueous solution. Many parameters, such as the structure, composition, size, and surface functional groups, have decisive roles in the QY of the NIR-II probe. For example, by using a core–shell strategy, lanthanide-based downconversion nanoparticles can achieve a higher QY. Therefore, it is necessary to develop NIR-II probes with high QDs by adjusting these parameters.

Finally, design and optimization of the NIR-II imaging equipment is also necessary. The conventional NIR-II optical imaging systems have the disadvantages of a small field of view, a low resolution and insufficient penetration depth. These drawbacks will limit the application of NIR-II optical imaging. Therefore, more efforts should be made to develop and optimize the function of the NIR-II imaging system.

## References

[1] Luo S, Zhang E, Su Y (2011). A review of NIR dyes in cancer targeting and imaging. Biomaterials.

[2] Peng Y, Xiong B, Peng L (2015). Recent advances in optical imaging with anisotropic plasmonic nanoparticles. Anal Chem.

[3] Hu X, Wang Q, Liu Y (2014). Optical imaging of articular cartilage degeneration using near-infrared dipicolylamine probes. Biomaterials.

[4] Chen G, Qiu H, Prasad PN (2014). Upconversion nanoparticles: design, nanochemistry, and applications in theranostics. Chem Rev.

[5] Zhang F, Zhao M, Wang R (2019). Precise in vivo inflammation imaging using in situ responsive cross-linking of glutathione-modified ultra-small NIR-II lanthanide nanoparticles. Angew Chem Int Ed Engl.

[6] Choy G, Choyke P, Libutti SK (2003). Current advances in molecular imaging: noninvasive in vivo bioluminescent and fluorescent optical imaging in cancer research. Mol Imaging.

[7] Erhan İA, Adair JH (2010). Near infrared imaging with nanoparticles. Wiley Interdiscipl Rev Nanomed Nanobiotechnol..

[8] Krumholz A, Shcherbakova DM, Xia J (2014). Multicontrast photoacoustic in vivo imaging using near-infrared fluorescent proteins. Sci Rep.

[9] Mishra A, Jiang Y, Roberts S (2016). Near-infrared photoacoustic imaging probe responsive to calcium. Anal Chem.

[10] Lian W, Tu D, Hu P (2020). Broadband excitable NIR-II luminescent nano-bioprobes based on CuInSe2 quantum dots for the detection of circulating tumor cells. Nano Today.

[11] Zhao J, Zhong D, Zhou S (2018). NIR-I-to-NIR-II fluorescent nanomaterials for biomedical imaging and cancer therapy. J Mater Chem B.

[12] Smith AM, Mancini MC, Nie S (2009). Bioimaging: second window for in vivo imaging. Nat Nanotechnol.

[13] Chen W, Xu S, Day JJ (2017). A general strategy for development of near-infrared fluorescent probes for bioimaging. Angew Chem Int Ed Engl.

[14] Miao Y, Gu C, Zhu Y (2018). Recent progress in fluorescence imaging of the near-infrared II window. ChemBioChem.

[15] Wang R, Li X, Zhou L, Zhang F (2014). Epitaxial seeded growth of rare-earth nanocrystals with efficient 800 nm near-infrared to 1525 nm short-wavelength infrared downconversion photoluminescence for in vivo bioimaging. Angew Chem Int Ed Engl.

[16] Wang P, Fan Y, Lu L (2018). NIR-II nanoprobes in-vivo assembly to improve image-guided surgery for metastatic ovarian cancer. Nat Commun.

[17] Chen G, Ohulchanskyy TY, Liu S (2012). Core/shell NaGdF4:Nd3+/NaGdF4 nanocrystals with efficient near-infrared to near-infrared downconversion photoluminescence for bioimaging applications. ACS Nano.

[18] Liu Z, Ren F, Zhang H (2019). Boosting often overlooked long wavelength emissions of rare-earth nanoparticles for NIR-II fluorescence imaging of orthotopic glioblastoma. Biomaterials.

[19] Zhao P, Xu Q, Tao J (2017). Near infrared quantum dots in biomedical applications: current status and future perspective. Wiley Interdiscip Rev Nanomed Nanobiotechnol..

[20] Du Y, Xu B, Fu T (2010). Near-infrared photoluminescent Ag2S quantum dots from a single source precursor. J Am Chem Soc.

[21] Zhang Y, Hong G, Zhang Y (2012). Ag2S quantum dot: a bright and biocompatible fluorescent nanoprobe in the second near-infrared window. ACS Nano.

[22] Zhao JY, Chen G, Gu YP (2016). Ultrasmall magnetically engineered Ag2Se quantum dots for instant efficient labeling and whole-body high-resolution multimodal real-time tracking of cell-derived microvesicles. J Am Chem Soc.

[23] Wu PJ, Kuo SY, Huang YC (2014). Polydiacetylene-enclosed near-infrared fluorescent semiconducting polymer dots for bioimaging and sensing. Anal Chem.

[24] Hong GS, Zou YP, Antaris AL (2014). Ultra-fast fluorescence imaging in vivo with conjugated polymer fluorophores in the second near-infrared window. Nat Commun.

[25] Reisch A, Klymchenko AS (2016). Fluorescent polymer nanoparticles based on dyes: seeking brighter tools for bioimaging. Small.

[26] Mao D, Liu J, Ji S (2017). Amplification of near-infrared fluorescence in semiconducting polymer nanoprobe for grasping the behaviors of systemically administered endothelial cells in ischemia treatment. Biomaterials.

[27] McDevitt MR, Scheinberg DA (2014). Fibrillous carbon nanotube: an unexpected journey. Crit Rev Oncog.

[28] Hong G, Diao S, Antaris AL (2015). Carbon nanomaterials for biological imaging and nanomedicinal therapy. Chem Rev.

[29] Yi H, Ghosh D, Ham MH (2012). M13 phage-functionalized single-walled carbon nanotubes as nanoprobes for second near-infrared window fluorescence imaging of targeted tumors. Nano Lett.

[30] Hong G, Dai H (2016). In vivo fluorescence imaging in the second near-infrared window using carbon nanotubes. Methods Mol Biol.

[31] Shangfeng W, Yong F (2019). Anti-quenching NIR-II molecular fluorophores for in vivo high-contrast imaging and pH sensing. Nat Commun..

[32] Huang J, Xie C, Zhang X (2019). Renal-clearable molecular semiconductor for second near-infrared fluorescence imaging of kidney dysfunction. Angew Chem Int Ed Engl.

[33] Kangquan S, Chunrong Q (2017). Multifunctional biomedical imaging in physiological and pathological conditions using a NIR-II probe. Adv Funct Mater..

[34] Tang Y, Li Y, Hu X (2018). "Dual lock-and-key"-controlled nanoprobes for ultrahigh specific fluorescence imaging in the second near-infrared window. Adv Mater.

[35] Zhang F, Li B, Lu L (2018). Efficient 1064-nm NIR-II excitation fluorescent molecular dye for deep-tissue high-resolution dynamic bioimaging. Angew Chem Int Ed Engl.

[36] He S, Chen S, Li D (2019). High affinity to skeleton rare earth doped nanoparticles for near-infrared II imaging. Nano Lett.

[37] Zhu S, Tian R, Antaris AL (2019). Near-infrared-II molecular dyes for cancer imaging and surgery. Adv Mater.

[38] Li CY, Wang QB (2018). Challenges and opportunities for intravital near-infrared fluorescence imaging technology in the second transparency window. ACS Nano.

[39] Welsher K, Liu Z, Sherlock SP (2009). A route to brightly fluorescent carbon nanotubes for near-infrared imaging in mice. Nat Nanotechnol.

[40] Welsher K, Sherlock SP, Dai H (2011). Deep-tissue anatomical imaging of mice using carbon nanotube fluorophores in the second near-infrared window. Proc Natl Acad Sci USA.

[41] He S, Song J, Qu J (2018). Crucial breakthrough of second near-infrared biological window fluorophores: design and synthesis toward multimodal imaging and theranostics. Chem Soc Rev..

[42] Diao S, Hong G, Robinson JT (2012). Chirality enriched (12,1) and (11,3) single-walled carbon nanotubes for biological imaging. J Am Chem Soc.

[43] Robinson JT, Hong G, Liang Y (2012). In vivo fluorescence imaging in the second near-infrared window with long circulating carbon nanotubes capable of ultrahigh tumor uptake. J Am Chem Soc.

[44] Li C, Cao L, Zhang Y (2015). Preoperative detection and intraoperative visualization of brain tumors for more precise surgery: a new dual-modality MRI and NIR nanoprobe. Small.

[45] Wu C, Zhang Y, Li Z (2016). A novel photoacoustic nanoprobe of ICG@PEG-Ag2S for atherosclerosis targeting and imaging in vivo. Nanoscale.

[46] Dong B, Li C, Chen G (2013). Facile synthesis of highly photoluminescent Ag2Se quantum dots as a new fluorescent probe in the second near-infrared window for in vivo imaging. Chem Mater.

[47] Zhao DH, Yang J, Xia RX (2018). High quantum yield Ag2S quantum dot@polypeptide-engineered hybrid nanogels for targeted second near-infrared fluorescence/photoacoustic imaging and photothermal therapy. Chem Commun.

[48] Zhao YX, Song ZM (2014). Phase transfer-based synthesis of highly stable, biocompatible and the second near-infrared-emitting silver sulfide quantum dots. Mater Lett.

[49] Liu B, Li C, Yang P (2017). 808-nm-Light-excited lanthanide-doped nanoparticles: rational design, luminescence control and theranostic applications. Adv Mater.

[50] Wang R, Zhou L, Wang W (2017). In vivo gastrointestinal drug-release monitoring through second near-infrared window fluorescent bioimaging with orally delivered microcarriers. Nat Commun.

[51] Naczynski DJ, Tan MC, Zevon M (2013). Rare-earth-doped biological composites as in vivo shortwave infrared reporters. Nat Commun.

[52] Wang R, Li X, Zhou L (2015). Epitaxial seeded growth of rare-earth nanocrystals with efficient 800 nm near-infrared to 1525 nm short-wavelength infrared downconversion photoluminescence for invivo bioimaging. Angew Chem Int Ed Engl.

[53] Yu M, Zheng J (2015). Clearance pathways and tumor targeting of imaging nanoparticles. ACS Nano.

[54] Yu J, Yin W, Peng T (2017). Biodistribution, excretion, and toxicity of polyethyleneimine modified NaYF4:Yb, Er upconversion nanoparticles in mice via different administration routes. Nanoscale.

[55] Chen H, Dong B, Tang Y (2017). A unique, "integration" strategy for the rational design of optically tunable near-infrared fluorophores. Acc Chem Res.

[56] Poronik YM, Vygranenko KV, Gryko D, Gryko DT (2019). Rhodols—synthesis, photophysical properties and applications as fluorescent probes. Chem Soc Rev.

[57] Jradi FM, Lavis LD (2019). Chemistry of photosensitive fluorophores for single-molecule localization microscopy. ACS Chem Biol.

[58] Song G, Zheng X, Wang Y, Xia X, Chu S, Rao J (2019). A magneto-optical nanoplatform for multimodality imaging of tumors in mice. ACS Nano.

[59] Wang Y, Shi L, Ye Z (2020). Reactive oxygen correlated chemiluminescent imaging of a semiconducting polymer nanoplatform for monitoring chemodynamic therapy. Nano Lett.

[60] Lu C, Zhang C, Wang P (2020). Light-free generation of singlet oxygen through manganese-thiophene nanosystems for pH-responsive chemiluminescence imaging and tumor therapy. Chem.

[61] Zhu H, Fang Y, Miao Q (2017). Regulating near-infrared photodynamic properties of semiconducting polymer nanotheranostics for optimized cancer therapy. ACS Nano.

[62] Tao Z, Hong G, Shinji C (2013). Biological imaging using nanoparticles of small organic molecules with fluorescence emission at wavelengths longer than 1000 nm. Angew Chem Int Ed.

[63] Hong G, Zou Y, Antaris AL, Diao S, Wu D, Cheng K, Zhang X, Chen C, Liu B, He Y, Wu JZ, Yuan J, Zhang B, Tao Z, Fukunaga C, Dai H (2014). Ultra-fast fluorescence imaging in vivo with conjugated polymer fluorophores in the second near-infrared window. Nat Commun..

[64] Antaris AL, Chen H, Cheng K (2016). A small-molecule dye for NIR-II imaging. Nat Mater.

[65] Kosaka N, Ogawa M, Choyke PL (2009). Clinical implications of near-infrared fluorescence imaging in cancer. Future Oncol.

[66] Skondra D, Papakostas TD, Hunter R (2012). Near infrared autofluorescence imaging of retinal diseases. Semin ophthalmol.

[67] Nguyen QT, Olson ES, Aguilera TA (2010). Surgery with molecular fluorescence imaging using activatable cell-penetrating peptides decreases residual cancer and improves survival. Proc Natl Acad Sci USA.

[68] Pysz MA, Gambhir SS, Willmann JK (2010). Molecular imaging: current status and emerging strategies. Clin Radiol.

[69] Hilderbrand SA, Weissleder R (2010). Near-infrared fluorescence: application to in vivo molecular imaging. Curr Opin Chem Biol.

[70] Vahrmeijer AL, Hutteman M, van der Vorst JR, van de Velde CJ, Frangioni JV (2013). Image-guided cancer surgery using near-infrared fluorescence. Nat Rev Clin Oncol.

[71] Andreou C, Neuschmelting V, Tschaharganeh DF (2016). Imaging of liver tumors using surface-enhanced Raman scattering nanoparticles. ACS Nano.

[72] Pereira ER, Kedrin D, Seano G (2018). Lymph node metastases can invade local blood vessels, exit the node, and colonize distant organs in mice. Science.

[73] Tian R, Ma H, Zhu S (2020). Multiplexed NIR-II probes for lymph node-invaded cancer detection and imaging-guided surgery. Adv Mater.

[74] Ramdasi S, Sarang S, Viswanathan C (2015). Potential of mesenchymal stem cell based application in cancer. Int J Hematol Oncol Stem Cell Res.

[75] Mimeault M, Hauke R, Batra SK (2007). Stem cells: a revolution in therapeutics-recent advances in stem cell biology and their therapeutic applications in regenerative medicine and cancer therapies. Clin Pharmacol Ther.

[76] Huang D, Lin S, Wang Q (2019). An NIR-II fluorescence/dual bioluminescence multiplexed imaging for in vivo visualizing the location, survival, and differentiation of transplanted stem cells. Adv Funct Mater.

[77] Yang Y, Chen J, Shang X (2019). Visualizing the fate of intra-articular injected mesenchymal stem cells in vivo in the second near-infrared window for the effective treatment of supraspinatus tendon tears. Adv Sci.

[78] Liu J, Luo Z, Zhang J (2016). Hollow mesoporous silica nanoparticles facilitated drug delivery via cascade pH stimuli in tumor microenvironment for tumor therapy. Biomaterials.

[79] Shen S, Chao Y, Dong Z (2017). Bottom-up preparation of uniform ultrathin rhenium disulfide nanosheets for image-guided photothermal radiotherapy. Adv Funct Mater.

[80] Shi B, Ren N, Gu L (2019). Theranostic nanoplatform with hydrogen sulfide activatable NIR responsiveness for imaging-guided on-demand drug release. Angew Chem Int Ed.

[81] Huang C, Sun Z, Cui H (2019). InSe nanosheets for efficient NIR-II-responsive drug release. ACS Appl Mater Interfaces.

[82] Li Y, Cai Z, Liu S (2020). Design of AIEgens for near-infrared IIb imaging through structural modulation at molecular and morphological levels. Nat Commun.

[83] Liu S, Chen C, Li Y (2020). Constitutional isomerization enables bright NIR-II AIEgen for brain-inflammation imaging. Adv Funct Mater.

[84] Wang S, Liu L, Fan Y (2019). In vivo high-resolution ratiometric fluorescence imaging of inflammation using NIR-II nanoprobes with 1550 nm emission. Nano Lett.

[85] Bashkatov AN, Genina EA, Kochubey VI (2005). Optical properties of human skin, subcutaneous and mucous tissues in the wavelength range from 400 to 2000nm. J Phys D Appl Phys.

[86] Jin Q, Zhu W, Jiang D (2017). Ultra-small iron-gallic acid coordination polymer nanoparticles for chelator-free labeling of ^64^Cu and multimodal imaging-guided photothermal therapy. Nanoscale.

[87] Ren F, Ding L, Liu H (2018). Ultra-small nanocluster mediated synthesis of Nd ^3+^ -doped core-shell nanocrystals with emission in the second near-infrared window for multimodal imaging of tumor vasculature. Biomaterials.

[88] Guo B, Chen J, Chen N (2019). High-resolution 3D NIR-II photoacoustic imaging of cerebral and tumor vasculatures using conjugated polymer nanoparticles as contrast agent. Adv Mater.

